# Assessment of Japanese iodine intake based on seaweed consumption in Japan: A literature-based analysis

**DOI:** 10.1186/1756-6614-4-14

**Published:** 2011-10-05

**Authors:** Theodore T Zava, David T Zava

**Affiliations:** 1 ZRT Laboratory, 8605 SW Creekside Place, Beaverton, 97008, USA

**Keywords:** Iodine, iodide, seaweed, algae, kelp, Japanese, thyroid, cancer, life expectancy

## Abstract

Japanese iodine intake from edible seaweeds is amongst the highest in the world. Predicting the type and amount of seaweed the Japanese consume is difficult due to day-to-day meal variation and dietary differences between generations and regions. In addition, iodine content varies considerably between seaweed species, with cooking and/or processing having an influence on iodine content. Due to all these factors, researchers frequently overestimate, or underestimate, Japanese iodine intake from seaweeds, which results in misleading and potentially dangerous diet and supplementation recommendations for people aiming to achieve the same health benefits seen by the Japanese. By combining information from dietary records, food surveys, urine iodine analysis (both spot and 24-hour samples) and seaweed iodine content, we estimate that the Japanese iodine intake--largely from seaweeds--averages 1,000-3,000 μg/day (1-3 mg/day).

## Introduction

Japanese iodine intake exceeds that of most other countries, primarily due to substantial seaweed consumption. Iodine is an essential element required for thyroid hormone synthesis, believed to impart some of its antioxidant and antiproliferative activity in the prevention of cardiovascular disease and cancer [1-8]. Seaweeds have the unique ability to concentrate iodine from the ocean, with certain types of brown seaweed accumulating over 30,000 times the iodine concentration of seawater [9]. The amount of iodine the Japanese consume daily from seaweeds has previously been estimated as high as 13.5 to 45 mg/day by sources that use ambiguous data to approximate intake [10,11], an amount 4.5 to 15 times greater than the safe upper limit of 3 mg/day set by the Ministry of Health, Labor and Welfare in Japan [12]. While high iodine intake from seaweed consumption is believed to have numerous health benefits, it has been reported to negatively affect individuals with underlying thyroid disorders [13-16]. To prevent excessive consumption it is imperative for people seeking health benefits from a high iodine diet to be knowledgeable of the amount of iodine the Japanese consume daily. In this paper we use a combination of dietary records, food surveys, urine iodine analysis, and seaweed iodine content to provide a reliable estimate of Japanese iodine intake, primarily from seaweeds.

## Types of edible seaweeds and their iodine content

In Japan, over 20 species of red, green, and brown algae (seaweed) are included in meals [17]. Iodine content varies depending on species, harvest location and preparation, and is typically highest in fresh cut blades and lowest in sun bleached blades [18]. The three most popular seaweed products in Japan are nori (*Porphyra*), wakame (*Undaria*) and kombu (*Laminaria*). Dried iodine contents range from 16 μg/g in nori to over 8,000 μg/g in kelp flakes; Japanese kombu and wakame contain an estimated 2353 μg/g and 42 μg/g respectively [18,19]. Ten different species of *Laminaria*, a type of kelp commonly labeled as kombu, from around the world were examined for their iodine content and were found to average 1,542 μg/g when dried [17].

## Japanese seaweed consumption statistics

As the Japanese transitioned from a traditional to a Westernized diet, beginning around the 1950's [20], consumption of certain seaweed species declined while others increased. A decrease in kombu consumption (844 to 685 g/year per household) and an increase in wakame consumption (727 to 1234 g/year per household) can be seen between the years of 1963 and 1973 [21]. Consumption of kombu per Japanese household dropped further to 450 g in 2006 (elders ate up to four times more than those under the age of 29) [19]. Since daily seaweed consumption per person in Japan has remained relatively consistent over the last 40 years (4.3 g/day in 1955 and 5.3 g/day in 1995) [22], it is believed that consumption of wakame and nori have made up for the decline in kombu consumption [23,24]. Both nori and wakame have relatively low iodine contents compared to kombu.

Seaweed consumption frequency differs from person to person in Japan, resulting in a constantly fluctuating iodine intake. Seaweed is served in approximately 21% of Japanese meals [25] with 20-38% of the Japanese male and female population aged 40-79 years consuming seaweed more than five times per week, 29-35% three to four times per week, 25-35% one to two times per week, 6-13% one to two times per month, and 1-2% rarely consuming seaweed [26]. A 2010 food frequency questionnaire on the Japanese Kombu Association website indicates that kelp (assuming kombu) is consumed at a rate of: 27.5% once per week, 25.5% once per month, 18% three or four times per week, and 15.9% once every few months, with only 6.1% of survey respondents stating they consume kelp nearly every day [27].

## Effect of cooking on seaweed iodine content

Seaweed is often cooked to flavor dishes or soup stocks before consumption. When kombu is boiled in water for 15 minutes it can lose up to 99% of its iodine content, while iodine in sargassum, a similar brown seaweed, loses around 40% [28,29]. Processed kelp is often boiled in dye for half an hour ("ao-kombu" or "kizami-kombu") before hanging to dry [21], a process which can reduce seaweed iodine content before it is consumed. When kelp is used to flavor soup stocks the seaweed is often removed after boiling, resulting in soup stock high in iodine. Twenty samples of supermarket soups with kelp or kelp broth were analyzed by Nishiyama et al. to determine iodine content, revealing a minimum concentration of 660 μg/L (0.66 mg/L) and a maximum concentration of 31,000 μg/L (31 mg/L) [16]. Serving size for soup is typically around 0.25 L, resulting in 165 to 7,750 μg (0.165 to 7.75 mg) of iodine per serving.

## Estimating Japanese iodine intake from seaweed consumption

Due to variation of iodine content from one seaweed species to the next, along with confusion stemming from wet and dry weight terminology, many inaccurate assumptions have been made regarding the amount of iodine the Japanese actually consume from seaweed. Not all studies, dietary records or surveys specify whether daily or yearly consumption of seaweeds is recorded using wet weight, dry weight or a combination of the two. In some reports seaweed consumption has been estimated at 4-7 g/day dried weight [17,22,30,31], while other reports claim consumption of 12 g/day using both wet and dry weight [32]. Certain seaweeds have a swelling capacity of nearly ten times their dry volume with moisture content typically over 70% when wet and around 7-20% when dried [33,34]. The difference between wet and dry weight, along with the type of seaweeds being consumed, can result in extreme overestimation (more likely) or underestimation (less likely) of Japanese iodine intake.

Interpreting information to determine Japanese seaweed consumption and resulting iodine intake is a difficult task, and with ever changing diets, a close estimate is all that can be made. Nori and wakame are the most commonly consumed seaweeds in Japan, with nori accounting for 45% and wakame accounting for 30% (75% together) of total seaweed consumption, as stated by the Food and Agriculture Organization of the United Nations [35]. Based on previous estimates and records, dried seaweed consumption of 4-7 g/day [17,22,30,31] results in iodine intakes between 79 and 139 μg/day from nori and wakame when calculated using dried iodine contents of 16 and 42 μg/g respectively [18]. The remainder of iodine intake is derived mainly from kombu consumption, with smaller amounts coming from other seaweeds that have nominal iodine content.

Kombu has the highest iodine content of all seaweeds in the Japanese diet. In 2006 consumption of kombu/household/year was 450 g [19], and with an average of 2.55 members per household in Japan in 2005 [36], 0.48 g kombu/person/day was consumed. When calculated, 0.48 g of kombu with an iodine content of 2,353 μg/g [18] equates to 1,129 μg/day of iodine. Assuming negligible iodine intake from the other seaweeds consumed, daily iodine intake from nori, wakame, and kelp can be estimated at 1,208 to 1,268 μg/day (1.2 to 1.3 mg/day). It is reasonable to assume that iodine intake per day based on seaweed consumption frequency and iodine content averages around 1,000-2,000 μg/day (1-2 mg/day).

## Estimating Japanese iodine intake from diet studies and urine iodine analysis

Seaweed consumption statistics only provide only an estimate of Japanese iodine intake and should be combined with other predictive factors. Fortunately, studies that measure iodine content of single or entire meals are available and are, arguably, the most accurate estimate of Japanese iodine intake from seaweeds. A collection of Japanese diet studies that measure the amount of iodine in 24-hour diet samples or single meals can be seen in Table 1. Daily iodine intake of the Japanese based on 24-hour diet samples generally does not exceed 3,000 μg (3 mg).

**Table 1:** Compilation of Japanese diet studies measuring iodine in 24-hour diet samples and single meals

Author(s) [source]	Year	Number of Participants	Mean Iodine in 24-Hour Diet Sample ( μg)	Lowest Iodine in 24-Hour Diet Sample ( μg)	Highest Iodine in 24-Hour Diet Sample ( μg)	City/Region
Katamine et al. [23]	1986	1	1023 μg	45 μg	1921 μg	Tokyo

Katamine et al. [23]	1986	1	362 μg	57 μg	1244 μg	Tokyo

Katamine et al. [23]	1986	1	361 μg	62 μg	1098 μg	Tokyo

Katamine et al. [23]	1986	1	429 μg	52 μg	1561 μg	Tokyo

Katamine et al. [23]	1986	10 (hospital)	1290 μg	89 μg	4746 μg	

Katamine et al. [23]	1986	5 (hospital)	195 μg	95 μg	287 μg	

Katamine et al. [23]	1986	13 (school)	113 μg/meal	47 μg/meal	203 μg/meal	Ibaraki

Katamine et al. [23]	1986	5 (school)	27 μg/meal	25 μg/meal	31 μg/meal	Kanagawa

Katamine et al. [23]	1986	5 (school)	36 μg/meal	18 μg/meal	43 μg/meal	Kanagawa

Tajiri et al. [48]	1986	1	25400 μg est.			Kumamoto

Tajiri et al. [48]	1986	1	43000 μg est.			Kumamoto

Tajiri et al. [48]	1986	1	15000 μg est.			Kumamoto

Tajiri et al. [48]	1986	1	20000 μg est.			Kumamoto

Tajiri et al. [48]	1986	10	2800 μg est.			Kumamoto

Tajiri et al. [48]	1986	8	2300 μg est.			Kumamoto

Shiraishi et al. [61]	1999	6	1770 μg	545 μg	4490 μg	Mito

Kunachowicz et al. [62]	2000	5	1970 μg/kg mean, 550 μg/kg median	88 μg/kg	7650 μg/kg	

Yoshinaga et al. [25]	2001	29 (476 meals)	1900 μg/kg			All Japan

Kucera et al. [63]	2003		756 μg/kg median	124 μg/kg	21660 μg/kg	

Nishiyama et al. [16]	2004	5 (pregnant)		2280 μg	3180 μg	Kumamoto

Nishiyama et al. [16]	2004	10 (pregnant)		820 μg	1400 μg	Kumamoto

Nishiyama et al. [16]	2004	22 (pregnant w/no kelp)		250 μg	480 μg	Kumamoto

Because approximately 97% of dietary iodine is excreted in the urine, urine iodine levels taken from individuals or populations can provide a secondary estimate of Japanese iodine intake from seaweed consumption, when paired with diet studies [37,38]. Urine iodine levels can increase from 100 μg/L to 30,000 μg/L in a single day and return to 100 μg/L within a couple of days, depending on seaweed intake [39]. This is somewhat expected when varying amounts and types of seaweeds are consumed on a day-to-day basis. Urine creatinine levels seen as μg iodine/g creatinine ( μg/g Cr) can be used to adjust for an individual's hydration status, correlating well with μg/L in areas of adequate nutrition [40]. Urine iodine levels of the Japanese found in a number of studies are shown in Table 2. Mean and median iodine levels in the Japanese urine collections typically do not exceed 3,000 μg/L (3 mg/L). When using 1.5 L as an expected 24-hour urine output, urine iodine excretion should rarely exceed an estimated 4,500 μg/24 hr (4.5 mg/24 hr).

**Table 2:** Compilation of Japanese urine iodine studies

Author(s) [source]	Year	Number of participants	Age	Sex	Mean Urine Iodine ( μg/L, μg/g Cr, or μg/24-hour)	Median Urine Iodine ( μg/L)	City/Region
Suzuki et al. [50]	1965	2			1565 μg/24-hour (hospital diet)		Hokkaido

Suzuki et al. [50]	1965	5			23300 μg/24-hour (seaweed diet)		Hokkaido

Suzuki et al. [50]	1965	7			175 μg/24-hour (iodine restricted)		Hokkaido

Nagataki et al. [39]	1967	9		Both	3286 μg/24-hour		Tokyo

Suzuki et al. [64]	1985	5	19-26	Male	357 μg/24-hour		

Suzuki et al. [64]	1985	10	19-21	Male	149 μg/24-hour		

Yabu et al. [65]	1986	127	18-57	Both	3238 μg/L		

Yabu et al. [66]	1988	127	18-57	Both	3022 μg/g Cr		

Yabu et al. [66]	1988	43	4-10	Both	2756 μg/g Cr		

Yabu et al. [66]	1988	30	Infant	Both	1854 μg/g Cr		

Yabu et al. [66]	1988	24	11-32	Female	1701 μg/g Cr		

Yabu et al. [66]	1988	73	18-27	Female	2845 μg/g Cr		

Nagataki [67]	1993	14			660 μg/g Cr		Tohoku

Nagataki [67]	1993	13			1090 μg/g Cr (hospital diet)		Tohoku

Nagataki [67]	1993	13			1760 μg/g Cr (hospital diet)		Tokyo

Nagataki [67]	1993	22			1460 μg/g Cr (hospital diet)		Shinsyu

Nagataki [67]	1993	8			1370 μg/g Cr (hospital diet)		Kyoto

Nagataki [67]	1993	19			910 μg/g Cr (hospital diet)		Nagasaki

Konno et al. [49]	1994	4138	Mean ~45	Both	3300 μg/L		Sapporo

Tsuda et al. [68]	1995	84				596 μg/L	Nagasaki

Nagata et al. [47]	1998	150	Mean ~52	Both	1480 μg/L		Nishihara

Nagata et al. [47]	1998	37			1470 μg/24-hour		Nishihara

Nagata et al. [47]	1998	20	Mean ~51	Both	1620 μg/L		Yamagata

Nagata et al. [47]	1998	54	Mean ~49	Both	1200 μg/L		Kobe

Nagata et al. [47]	1998	80	Mean ~50	Both	810 μg/L		Hotaka

Ishigaki et al. [69]	2001	250	7-14	Both		362 μg/L	Nagasaki

Ishigaki et al. [69]	2001	50	Adult	Both		208 μg/L	Hamamatsu

Ishigaki et al. [69]	2001	50	Adult	Both		1015 μg/L	South Kayabe

Takamura et al. [70]	2003	4	18-24	Male	406 μg/L		Nagasaki

Zimmermann et al. [71]	2005	302	6-12	Both	296 μg/L	292 μg/L	Central Hokkaido

Zimmermann et al. [71]	2005	280	6-12	Both	728 μg/L	741 μg/L	Costal Hokkaido

Tomoda et al. [72]	2005	47	Mean ~53	Both	428 μg/g Cr		

Tomoda et al. [72]	2005	21	Mean ~56	Both	587 μg/g Cr		

Fuse et al. [73]	2007	654	6-12	Both		281 μg/L	Tokyo

Miyai et al. [74]	2008	6	Mean ~27	Both	560 μg/24-hour		

Miyai et al. [74]	2008	14	Mean ~27	Both	1110 μg/24-hour		

Orito et al. [75]	2009	514	Adult	Female (pregnant)		328 μg/L	Kobe

## Japanese health statistics linked to high seaweed intake

The Japanese are considered one of the world's longest living people, with an extraordinarily low rate of certain types of cancer. A major dietary difference that sets Japan apart from other countries is high iodine intake, with seaweeds the most common source. Here are some astonishing Japanese health statistics, which are possibly related to their high seaweed consumption and iodine intake:

-Japanese average life expectancy (83 years) is five years longer than US average life expectancy (78 years) [41].

-In 1999 the age-adjusted breast cancer mortality rate was three times higher in the US than in Japan [42].

-Ten years after arriving in the US (in 1991), the breast cancer incidence rate of immigrants from Japan increased from 20 per 100,000 to 30 per 100,000 [43].

-In 2002 the age-adjusted rate of prostate cancer in Japan was 12.6 per 100,000, while the US rate was almost ten times as high [44].

-Heart related deaths in men and women aged 35-74 years are much higher in the US (1,415 per 100,000) as they are in Japan (897 per 100,000) [45].

-In 2004, infant deaths were over twice as high in the US (6.8 per 1,000) as they were in Japan (2.8 per 1,000) [46].

## Negative effects of iodine from seaweed

High iodine intake from seaweed consumption can cause unexpected health problems in a subset of individuals with pre-existing thyroid disorders. Although it is reported that excessive iodine does not cause thyroid antibody positivity, high intake can cause or worsen symptoms for people with previous thyroid autoimmunity or other underlying thyroid issues [47]. Transient hypothyroidism and iodine-induced goiter is common in Japan and can be reversed in most cases by restricting seaweed intake [16,29,48-52]. In Asian cultures, seaweed is commonly cooked with foods containing goitrogens such as broccoli, cabbage, bok choi and soy [18]. The phytochemicals in these foods can competitively inhibit iodine uptake by the thyroid gland (i.e., isothiocyanates from cruciferous vegetables) [53-55], or inhibit incorporation of iodine into thyroid hormone (i.e., soy isoflavones) [56,57].

Certain species of seaweed can concentrate bromine, a halide similar to iodine with no known physiological function, at very high levels [58,59]. If seaweeds with elevated levels of bromine and low levels of iodine are consumed when the body is in an iodine deficient state, inhibition of thyroid hormone synthesis--due to bromine's attachment to tyrosine residues on thyroglobulin in place of iodine--is plausible [60].

## Estimate of daily iodine intake in Japan

We estimate that the average Japanese iodine intake, largely from seaweed consumption--based on dietary records, food surveys, urine iodine analysis and seaweed iodine content--is 1,000-3,000 μg/day (1-3 mg/day). This estimate compares to a recent report claiming that the average iodine intake of the Japanese from kelp is around 1,200 μg/day (1.2 mg/day) [19]. Iodine intake can vary from day-to-day depending on diet, and it is unlikely for a single persons iodine intake to remain constant for an extended period of time. With the multitude of edible seaweeds (each with different iodine content) consumed in the Japanese diet, it is not appropriate to use a single type of seaweed to determine iodine intake, though many estimates do. Although seaweed provides a majority of the Japanese iodine intake, other food sources (containing far less iodine)--such as fish and shellfish--can increase the total amount of iodine consumed daily.

## Conclusions

Japanese iodine intake from seaweed is linked to health benefits not seen in cultures with dissimilar diets. Knowing how much iodine the Japanese consume daily is beneficial for people who wish to consume equivalent amounts of iodine or seaweed supplements while avoiding excessive amounts that may adversely affect health.

## Abbreviations

Cr: Creatinine.

## Competing interests

The author declares that they have no competing interests.

## Authors' contributions

TZ acquired and compiled data shown in this study, interpreted data, and provided intellectual content. DZ provided interpretation of data and intellectual content. All authors read and approved the final manuscript.

## Authors' information

TZ received his Bachelor's degree in Biology from Oregon State University in 2009. He is a Research Associate at ZRT Laboratory in Beaverton, Oregon, where he recently developed a test to measure iodine and creatinine levels in dried urine. His current research focuses on iodine deficiency, Japanese iodine intake, halide competition, thyroid disorders and iodine kinetics in the human body.

DZ received his doctorate in Biochemistry from the University of Tennessee in 1974. He is Laboratory Director and President of ZRT Laboratory, which he founded in 1998. Dr. Zava has developed innovative, simple and cost-effective methods to monitor hormone and other analytes associated with health and disease. His current research focus includes endocrinology, breast cancer, and--most recently--the importance of iodine to optimum health. He is co-author of a landmark book, What Your Doctor May Not Tell You About Breast Cancer: How Hormone Balance Can Help Save Your Life.
